# Contribution of NADPH Oxidase to Membrane CD38 Internalization and Activation in Coronary Arterial Myocytes

**DOI:** 10.1371/journal.pone.0071212

**Published:** 2013-08-07

**Authors:** Ming Xu, Xiao-Xue Li, Joseph K. Ritter, Justine M. Abais, Yang Zhang, Pin-Lan Li

**Affiliations:** Department of Pharmacology and Toxicology, Virginia Commonwealth University School of Medicine, Richmond, Virginia, United States of America; Tohoku University, Japan

## Abstract

The CD38-ADP-ribosylcyclase-mediated Ca^2+^ signaling pathway importantly contributes to the vasomotor response in different arteries. Although there is evidence indicating that the activation of CD38-ADP-ribosylcyclase is associated with CD38 internalization, the molecular mechanism mediating CD38 internalization and consequent activation in response to a variety of physiological and pathological stimuli remains poorly understood. Recent studies have shown that CD38 may sense redox signals and is thereby activated to produce cellular response and that the NADPH oxidase isoform, NOX1, is a major resource to produce superoxide (O_2_^·**−**^) in coronary arterial myocytes (CAMs) in response to muscarinic receptor agonist, which uses CD38-ADP-ribosylcyclase signaling pathway to exert its action in these CAMs. These findings led us hypothesize that NOX1-derived O_2_^·**−**^ serves in an autocrine fashion to enhance CD38 internalization, leading to redox activation of CD38-ADP-ribosylcyclase activity in mouse CAMs. To test this hypothesis, confocal microscopy, flow cytometry and a membrane protein biotinylation assay were used in the present study. We first demonstrated that CD38 internalization induced by endothelin-1 (ET-1) was inhibited by silencing of NOX1 gene, but not NOX4 gene. Correspondingly, NOX1 gene silencing abolished ET-1-induced O_2_^·**−**^ production and increased CD38-ADP-ribosylcyclase activity in CAMs, while activation of NOX1 by overexpression of Rac1 or Vav2 or administration of exogenous O_2_^·**−**^ significantly increased CD38 internalization in CAMs. Lastly, ET-1 was found to markedly increase membrane raft clustering as shown by increased colocalization of cholera toxin-B with CD38 and NOX1. Taken together, these results provide direct evidence that Rac1-NOX1-dependent O_2_^·**−**^ production mediates CD38 internalization in CAMs, which may represent an important mechanism linking receptor activation with CD38 activity in these cells.

## Introduction

CD38, first identified as a leukocyte differentiation antigen, is a type II transmembrane glycoprotein comprised of a short N-terminal cytoplasmic domain, a hydrophobic transmembrane region, and a long C-terminal extracellular domain [1]. In signaling, CD38 functions as a multifunctional enzyme that uses NAD^+^ or NADP^+^ as substrate to produce cyclic ADP-ribose (cADPR) or nicotinic acid adenine dinucleotide phosphate (NAADP), two potent Ca^2+^ mobilizers [2]. Recent studies in our laboratory and by others have reported that in vascular smooth muscle cells, cADPR contributes to Ca^2+^ release from the sarcoplasmic reticulum (SR) induced by inositol 1,4,5-trisphosphate (IP3)-independent agonists, such as acetylcholine, oxotremorine (Oxo), 5-hydroxytryptamine, angiotensin II (Ang II), and endothelin, leading to the contraction of arterial smooth muscle via ryanodine receptor (RyR)-mediated intracellular Ca^2+^ release [3], [4], [5]. NAADP is the newly difined and most potent intracellular universal Ca^2+^ messenger [6], which has also been shown to participate in the regulation of vasoconstriction [7]. Accumulating evidence has demonstrated that NAADP mediates a two-phase Ca^2+^ release response where NAADP mobilizes Ca^2+^ from a thapsigargin-insensitive lysosome-like acidic store to produce a local spatial Ca^2+^ signal, which triggers Ca^2+^-induced Ca^2+^ release (CICR) to cause global Ca^2+^ increases through RyRs on the SR [8], [9]. These cADPR/RyR and NAADP-mediated Ca^2+^ signaling pathways are now recognized as fundamental mechanisms regulating vascular function. However, the molecular mechanism of CD38 activation remains largely unknown.

Accumulating evidence indicates that CD38 activity is stimulated through cell-surface signals via activation of heterotrimeric G-protein-coupled receptors including β-adrenergic, Ang II, and muscarinic receptors [10]. Upon stimulation, CD38 was reported to undergo a ligand-induced, vesicle-mediated internalization in a variety of cells including B-lymphocytic and human Namalwa cells [11], [12]. CD38 topologic analysis explains the functionality of its internalization since the enzymatic active site of CD38 is located in the extracellular domain while the substrates (NAD^+^ or NADP^+^) for CD38 activity are present in the cytoplasm and thus CD38 internalization may expose its active site to the substrates. Although it is still unclear how CD38 in the internalized vesicles produces Ca^2+^ mobilizing messengers to exert its signaling action, CD38 internalization has been considered as a crucial step for CD38 activation [13], [14]. To date, the molecular mechanism regulating CD38 internalization is not completely understood.

Recent studies in our laboratory and by others have demonstrated an important mechanism mediating the redox regulation of CD38 activity [15]. It has been shown that enhanced oxidative stress or thiol compounds increase both CD38 activity and Ca^2+^ signaling, possibly via disulfide bond formation [14]. NADPH oxidase has been reported to be a major source of reactive oxygen species (ROS) in the vasculature [16]. Interestingly, NAD^+^ and NADP^+^, substrates for CD38, are the side products of NADPH oxidase. In addition to superoxide (O_2_^·**−**^), NADP^+^ was reported to induce CD38 internalization [11], [12]. Thus, NADPH oxidase may play dual roles in CD38 internalization via redox regulation and NADP^+^-mediated mechanisms. More recently, we have demonstrated that in coronary arterial myocytes (CAMs), NOX1 and NOX4 are two important NADPH oxidase isoforms, respectively, responsible for extracellular and intracellular O_2_^·**−**^ production [17]. Whereas, NOX2 was not found to have any significant effect on extracellular and intracellular O_2_^·**−**^ production in response to Ang II or oxotremorine in CAMs [15], [17], [18]. The topological characteristic of NOX1-derived extracellular O_2_^·**−**^ has led us to wonder whether this extracellular O_2_^·**−**^ could serve in an autocrine fashion to enhance CD38 internalization and consequent redox activation of this ecto-enzyme.

In the present study, we used a series of molecular and physiological approaches to test this hypothesis. We first determined if NOX1-derived O_2_^·**−**^ production contributes to receptor-coupled CD38 internalization and activation in mouse CAMs. Then, we examined whether activation of NOX1 by overexpression of its activator, Rac1 GTPase also causes CD38 internalization in these cells. Finally, we determined whether lipid rafts (currently named as membrane raft (MR)) clustering mediates CD38-NOX1 interaction leading to CD38 internalization and activation.

## Materials and Methods

### Mice

Mice were purchased from the Jackson Laboratory. Eight-week old male and female mice were used in all experiments. All experimental protocols were reviewed and approved by the Institutional Animal Care and Use Committee of Virginia Commonwealth University.

### Isolation and Culture of Mouse CAMs

CAMs were isolated from mice as previously described [19]. In brief, mice were deeply anesthetized with an intraperitoneal injection of pentobarbital sodium (25 mg/kg). The heart was excised with an intact aortic arch and immersed in a petri dish filled with ice-cold Krebs-Henseleit (*KH*) solution (in mM 20 HEPES, 128 NaCl, 2.5 KCl, 2.7 CaCl_2_, 1 MgCl_2_, 16 glucose, pH 7.4). A 25-gauge needle filled with Hanks’ buffered saline solution (HBSS) (in mM: 5.0 KCl, 0.3 KH_2_PO_4_, 138 NaCl, 4.0 NaHCO_3_, 0.3 Na_2_HPO_4_·7 H_2_O, 5.6 D-glucose, and 10.0 HEPES, with 2% antibiotics) was inserted into the aortic lumen opening while the whole heart remained in the ice-cold buffer solution. The opening of the needle was inserted deep into the heart close to the aortic valve. The needle was tied in place with the needle tip as close to the base of the heart as possible. The infusion pump was started with a 20-ml syringe containing warm HBSS through an intravenous extension set at a rate of 0.1 ml/min for 15 min. HBSS was replaced with a warm enzyme solution (1 mg/ml collagenase type I, 0.5 mg/ml soybean trypsin inhibitor, 3% BSA, and 2% antibiotic-antimycotic), which was flushed through the heart at a rate of 0.1 ml/min. Perfusion fluid was collected at 30-, 60-, and 90-min intervals. At 90 min, the heart was cut with scissors and the apex was opened to flush out the cells that collected inside the ventricle. The fluid was centrifuged at 1,000 rpm for 10 min, the cell-rich pellets were mixed with the one of the media described below, and the cells were plated on 2% gelatin-coated six-well plates and incubated in 5% CO_2_–95% O_2_ at 37°C. Advanced DMEM with 10% FBS, 10% mouse serum, and 2% antibiotics was used for isolated smooth muscle cells. The medium was replaced three days after cell isolation and then once or twice each week until the cells grew to confluence. As previously described [17], mouse CAMs were identified according to their morphology, immunohistological staining, Western blot analysis of marker proteins, and flow cytometric characteristics.

### RNA Interference

Small interference RNAs (siRNAs) were commercially available (QIAGEN, CA), and the sequences were as follows: NOX1 siRNA: 5′-UGGAGUCACUCCAUUUGCAUCGAUA-3′; NOX4 siRNA: 5′-UUUAGGGACAGCCAAAUGAGCAGGC-3′. NOX1 and NOX4 siRNA have been demonstrated to efficiently inhibit the protein expression of NOX1 and NOX4 as we recently described [17]. The scrambled small RNA (5′-AAUUCUCCGAACGUGUCACGU-3′) was also confirmed as non-silencing double stranded RNA and used as a control in the present study. In addition, Rac1 cDNA and Vav2 cDNA were also used in our previous study [20], and their effects were further confirmed with Western blot in the present study. Transfection of siRNA or cDNA was performed using the siLentFect Lipid Reagent or TransFectin Lipid Reagent (Bio-Rad, CA, USA) according to the manufacturer’s instructions.

### Confocal Microscopy of Internalized CD38

After treatment, CAMs were fixed with 4% paraformaldehyde in phosphate-buffered saline (PBS) and incubated at 4°C for 1 h and then washed three times with ice-cold PBS. The cells were permeabilized with 0.1% Triton X-100 in PBS at 4°C for 30 min and incubated with goat anti-CD38 mAb (1∶200; Santa Cruz Biotechnology, CA), followed by Alexa 488-conjugated anti-goat secondary antibody (1∶500, Molecular Probes, Carlsbad, CA) in the presence of 0.1% BSA at 4°C for 8 h. After mounting, the internalization was visualized by a confocal laser scanning microscope (Fluoview FV1000, Olympus, Japan). In addition, positive cells with internalized CD38 were counted and the percentage of positive cells was calculated.

### Flow-cytometric Analysis of CD38 Expression on the Cell Membrane

The expression of CD38 on the cell membrane was also assessed by flow cytometry. As described previously [21], CAMs were harvested and washed with PBS, and then blocked with 1% BSA for 10 min at 4°C. Cell viability, assessed by trypan blue staining, was always greater than 96%. After two washes, the pellet was added to 100 µl PBS and incubated with goat anti-CD38 IgG (1∶200), followed by incubation with Alex488-labeled rabbit anti-goat secondary antibody (BD Biosciences; 1∶500). Stained cells were run on a Guava Easycyte Mini Flow Cytometry System (Guava Technologies, Hayward, CA) and analyzed with Guava acquisition and analysis software (Guava Technologies).

### Cell Surface Biotinylation Assay for Expression of CD38 Protein

As described previously [22], after treatment, CAMs (1×10^6^ cells/well) were washed twice with PBS and then incubated twice with 0.5 mg/ml Sulfo-NHS-LC-Biotin in DMEM without serum for 10 min at 4°C. After washing with serum-free DMEM for 10 min and three times with PBS for 5 min at 4°C, cells were solubilized in lysis buffer (10 mM Tris, 150 mM NaCl, 1 mM EDTA, 0.1% SDS, 1% Triton X-100 with protease inhibitor mixture, pH 7.4) for 30 min at 4°C. Lysates were then centrifuged for 5 min at 1,500×*g*, and streptavidin beads were added to the supernatant to isolate cell membrane proteins. After incubation of the mixture for 16 h at 4°C, biotin-streptavidin beads complexes were sedimented at 13,000 rpm for 3 min. The supernatant was used as control and after two washes with PBS, bead-bound proteins were denatured in Laemmli buffer and analyzed by SDS-PAGE followed by Western blotting. Transferrin receptor (TIR) was a conserved membrane protein used as a loading control in this study. Cell surface exposure of protein was normalized to 25 µg of total lysate for each sample.

### Electron Spin Resonance (ESR) Detection of O_2_^·**−**^

For detection of the O_2_^·**−**^ production dependent on NADPH oxidase in the membrane, CAMs were gently collected and resuspended in modified Krebs-Hepes buffer containing deferoximine (100 µmol/L; Sigma, St. Louis, MO, USA) and diethyldithiocarbamate (5 µmol/L; Sigma). These mixtures containing 1×10^6^ cells were subsequently mixed with 1 mM of the O_2_^·**−**.^-specific spin trap, 1-hydroxy-3- methoxycarbonyl-2,2,5,5-tetramethylpyrrolidine (CMH) in the presence or absence of manganese-dependent superoxide dismutase (SOD) (200 U/ml; Sigma). The mixtures were then loaded into glass capillaries and immediately analyzed for O_2_^·**−**^ formation kinetics for 10 min in a Miniscope MS200 ESR spectrometer (Magnettech Ltd., Berlin, Germany) as described [23]. The ESR settings were as follows: biofield, 3350; field sweep, 60 G; microwave frequency, 9.78 GHz; microwave power, 20 mW; modulation amplitude, 3 G; 4096 points of resolution; receiver gain, 50 for cells. The results were expressed as the fold change of the treatment group versus the control.

### HPLC Analysis of NAADP Conversion Rate in CAMs

To determine CD38-associated NAADP production, we measured base-exchange-related NAADP conversion using HPLC in CAMs. Within a 100 µL reaction mixture, 1 mM NADP^+^ and 30 mM nicotinic acid (NA) as substrates were added to 100 µg of cell homogenates in HEPES buffer containing (in mM) 20 HEPES, 1 EDTA, and 255 sucrose (pH 4.5). After incubation at 37°C for 30 min, the proteins were removed by centrifugation using an Amicon microultrafilter at 13,000 rpm for 15 min. The reaction product in the ultrafiltrate was then analyzed using HPLC as described previously [7]. Peak identities were confirmed by comigration, and absorbance spectra were compared with the known standards. Quantitative measurements were performed by comparison of known concentrations of standards.

### Confocal Microscopy of the Colocalization of MR Clusters and NOX1 or CD38

For dual-staining detection of the colocalization of MR marker with NOX1 or CD38, the CAMs were first incubated with Alexa488-Cholera-toxin B (Al488-CtxB), as described previously [24], and then as needed, with rat anti-NOX1 IgG antibody (1∶200; Abcam, MA) followed by Texas red-conjugated anti-rat secondary antibody (Molecular Probes, Eugene, OR), or goat anti-CD38 mAb (1∶200; Santa Cruz Biotechnology, CA) followed by Texas red-conjugated anti-goat secondary antibody. Then, the colocalizations were visualized by confocal microscopic analysis. In addition, CtxB clusters positive cells were counted from total 50 cells under fluorescent microscope. Then, the percent changes of CtxB clusters positive cells were calculated.

### Statistics

Data are presented as means ± SE. Significant differences between and within multiple groups were examined using ANOVA for repeated measures, followed by Duncan’s multiple-range test. The Students *t* test was used to detect significant differences between two groups. P<0.05 was considered statistically significant.

## Results

### CD38 Internalization in CAMs before and after Silencing of NOX1 or NOX4 Gene

**Figure 1:**
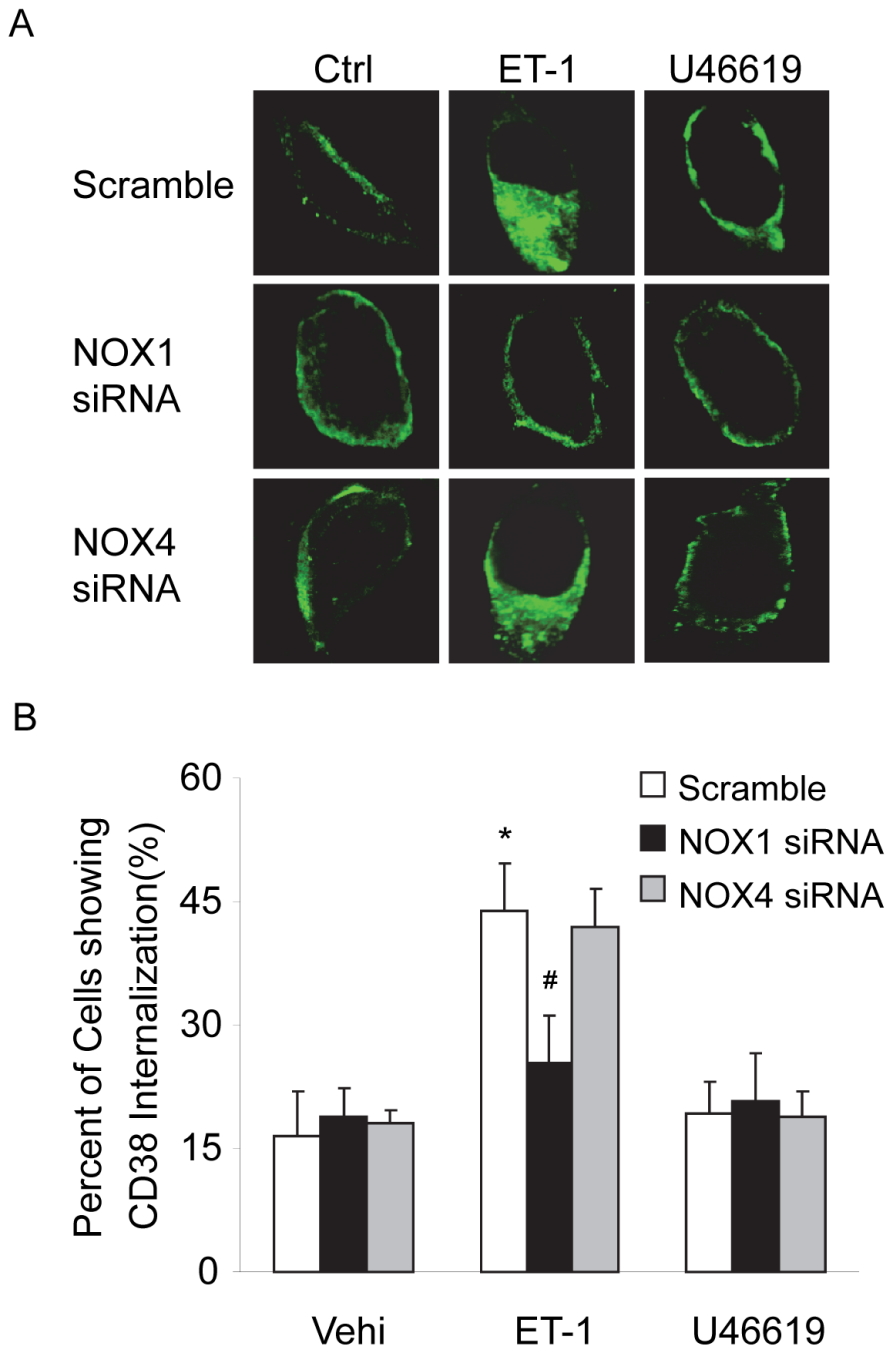
NOX1 mediated ET-1-induced CD38 internalization in CAMs. Mouse CAMs were treated with ET-1(100 nM) or U46619 (20 nM) for 10 min with or without transfection of NOX1 siRNA or NOX4 siRNA. Cells were then fixed, permeablized and stained with Alexa488 conjugated anti-CD38 antibodies. The expression of CD38 proteins in the cytosol was analyzed by confocal fluorescent microscopy. (A) Representative confocal fluorescent images of CD38 showing ET-1 but not U46619 induced CD38 internalization in mouse CAMs. (B) Summarized data showing the effects of NOX1 or NOX4 gene silencing on ET-1-induced CD38 internalization in CAMs. *P<0.05 *vs.* scramble control; ^#^ *P*<0.05 *vs.* scramble ET-1 (n = 6).

**Figure 2:**
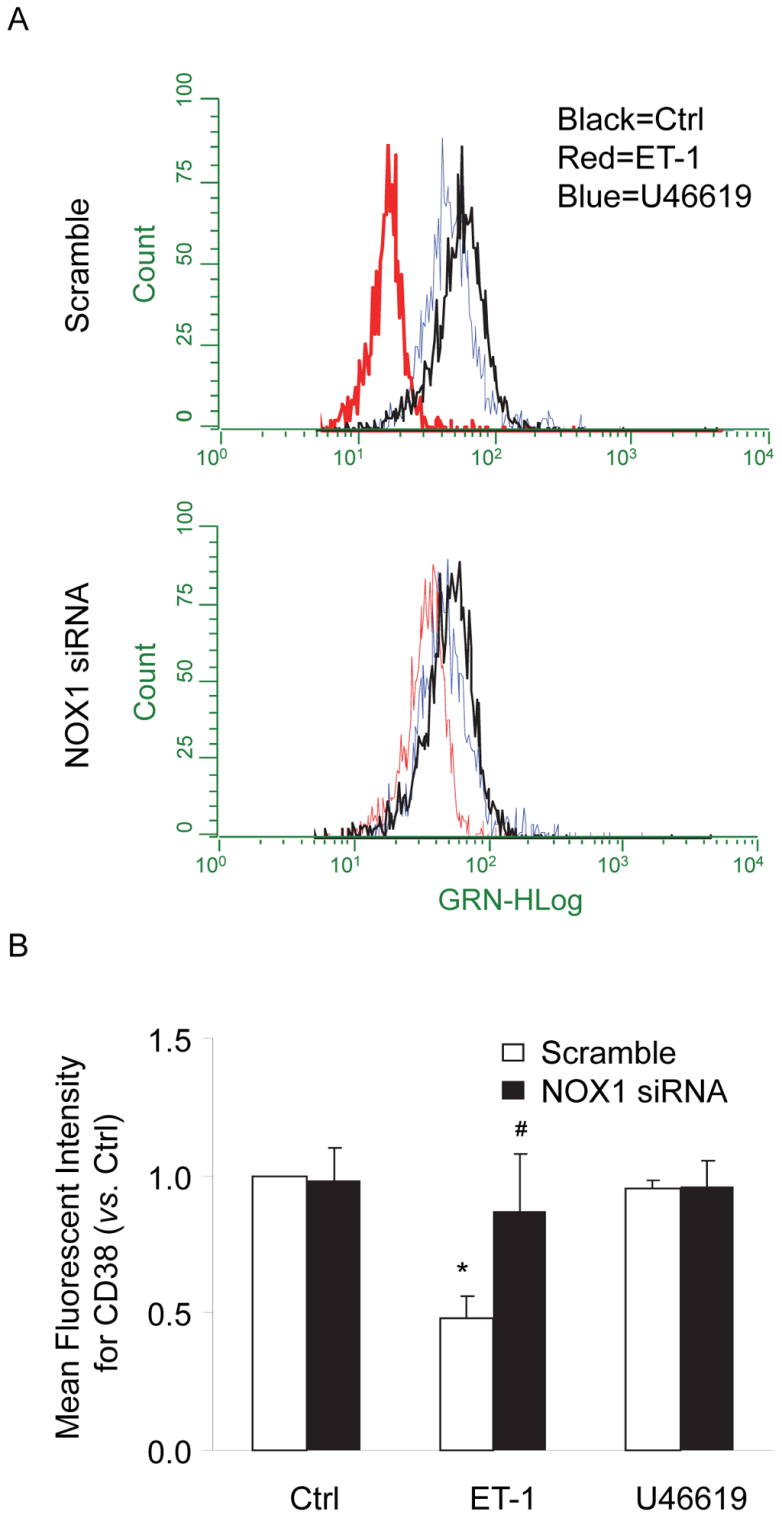
Flow cytometric analysis of sureface CD38 expression in living CAMs. Mouse CAMs were treated with ET-1(100 nM) or U46619 (20 nM) for 10 min with or without transfection of NOX1 siRNA. Cells were then stained with Alexa488 conjugated anti-CD38 antibodies without fixation and permeablization. The expression of CD38 proteins in these live cells were analyzed by flow cytometry. (A) Representative flow cytometry analysis from six independent experiments. (B) Summarized data showing the mean fluorescent intensity for Alexa488-anti-CD38 staining. *P<0.05 *vs.* scramble control; ^#^ *P*<0.05 *vs.* scramble ET-1 (n = 6).

**Figure 3:**
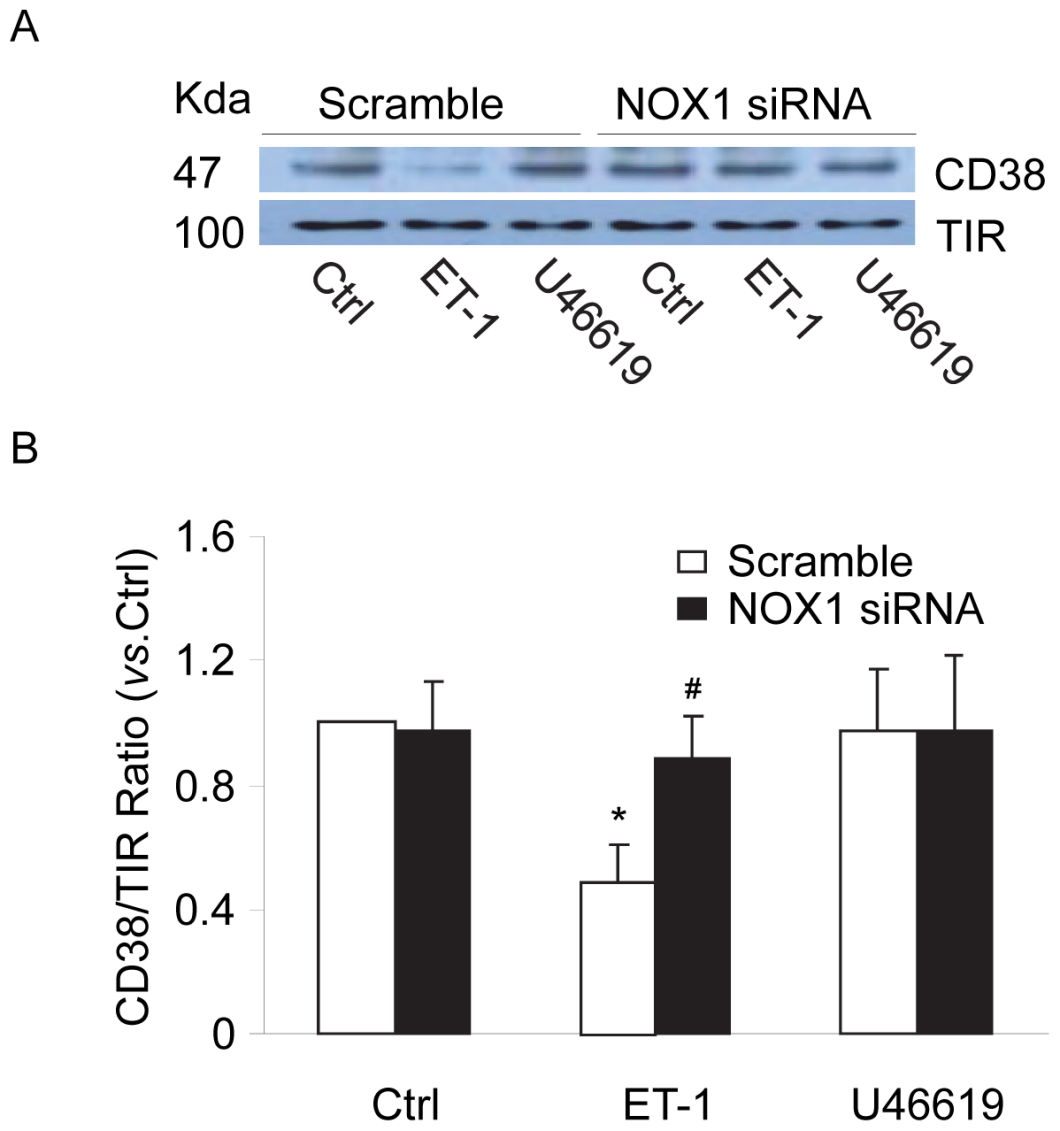
NOX1 activation by ET-1 decreased CD38 in the plasma membrane measured by biotinylated trapping. Mouse CAMs were treated with ET-1(100 nM) or U46619 (20 nM) with or without transfection of NOX1 siRNA. The proteins in the plasma membrane of these cells were biotinylated, precipitated by streptavidin beads and analyzed by Western blot. (A) Representative Western blot gel document showing the expression of CD38 and a plasma membrane marker, transferrin receptor (TIR), in the plasma membrane of CAMs. (B) Summarized data of Western blot. *P<0.05 *vs.* scramble control; ^#^ *P*<0.05 *vs.* scramble ET-1 (n = 5).

We first detected CD38 internalization in CAMs by confocal microscopic analysis of cytosolic expression of CD38 using Alexa 488-conjugated anti-CD38 antibodies. As shown in Figure 1A, under control conditions, CD38 staining was primarily observed around the edges of cells indicating the localization of this protein in the plasma membrane. Stimulation of CAMs with ET-1, a CD38 agonist, significantly increased CD38 localization in the peri-nuclear region of the cells, which is indicative of redistribution of CD38 proteins from the plasma membrane into cytosol, i.e. CD38 internalization in these cells. We also demonstrated that this ET-1-induced CD38 internalization was markedly attenuated in NOX1 siRNA, but not NOX4 siRNA-transfected CAMs. However, U46619, an inositol 1,4,5-trisphosphate (IP_3_) receptor-mediated Ca^2+^ release agonist had no effect on CD38 internalization in CAMs. To quantify CD38 internalization, the percentage of cells showing cytosolic expression of CD38 was calculated and summarized in Figure 1B, demonstrating that ET-1 increased CD38 internalization in a NOX1-dependent manner.We further investigated whether ET-1 induces CD38 internalization by examining the surface expression of CD38 proteins in living CAMs using flow cytometry. As shown in Figure 2A and 2B, the results demonstrated that ET-1 but not U46619 induced significant decreases in surface CD38 staining of CAMs. Such ET-1-induced decreases in surface CD38 staining were not observed in CAMs with NOX1 gene silencing. To further quantify the changes in the CD38 expression in the plasma membrane, proteins were biotinylated on the plasma membrane and then pulled down by streptavidin beads and analyzed by Western blot. As shown in Figure 3A and 3B, the intensity ratio of CD38 to transferrin receptor (TIR, a plasma membrane marker) decreased by almost half when CAMs were treated with ET-1, suggesting that 47.4% of the CD38 protein in the plasma membrane was internalized upon ET-1 stimulation. However, ET-1 did not decrease the CD38 expression in the plasma membrane when CAMs were first transfected with NOX1 siRNA. Consistently, U46619 had no effects on CD38 expression in the plasma membrane of CAMs.

### NOX1 Required for ET-1-induced O_2_^·**−**^ Production in CAMs

**Figure 4:**
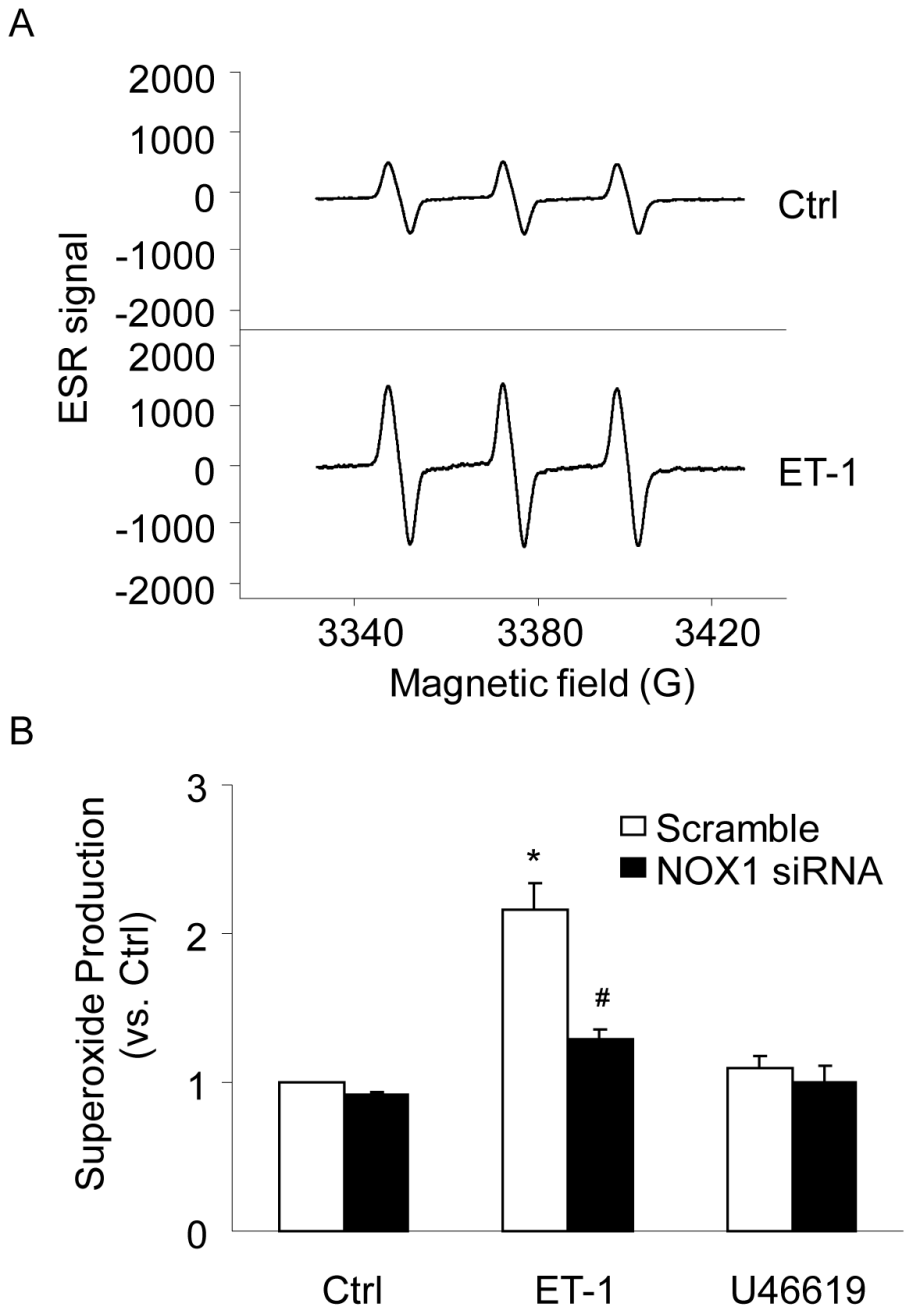
Effects of NOX1 gene silencing on ET-1-induced O_2_^·−^ production in CAMs. (A) Representative ESR spectrographs of O_2_ **^·−^** trapped by a spin probe CMH. (B) Summarized data showing the effects of ET-1 or U46619 on O_2_ **^·−^** production in CAMs transfected with scramble or NOX1 siRNA. *P<0.05 *vs.* scramble control; ^#^ *P*<0.05 *vs.* scramble ET-1 (n = 6).

To examine whether NOX1 is indeed activated by ET-1 in CAMs, we directly measured O_2_^·**−**^ production using ESR spectrometry. In these experiments, SOD-sensitive signals in cell suspensions were measured to represent extracellular NADPH-dependent O_2_^·**−**^. Although this ESR spectrometric assay could not be used to detect O_2_^·**−**^ levels from a single cell [25], it is important that the signal, which is highly specific to O_2_^·**−**^, can be detected outside CAMs. Figure 4A shows representative changes in the SOD-inhibitable ESR spectrometric curve recorded under control conditions and after ET-1 stimulation. The summarized data demonstrated that ET-1 significantly increased production of SOD-sensitive O_2_^·**−**^ in CAMs, which was markedly attenuated by knockdown of NOX1 (Figure 4B). In contrast, U46619 had no significant effect on O_2_^·**−**^ production in CAMs.

### Activation of NOX1 Enhanced CD38 Activity in CAMs

**Figure 5:**
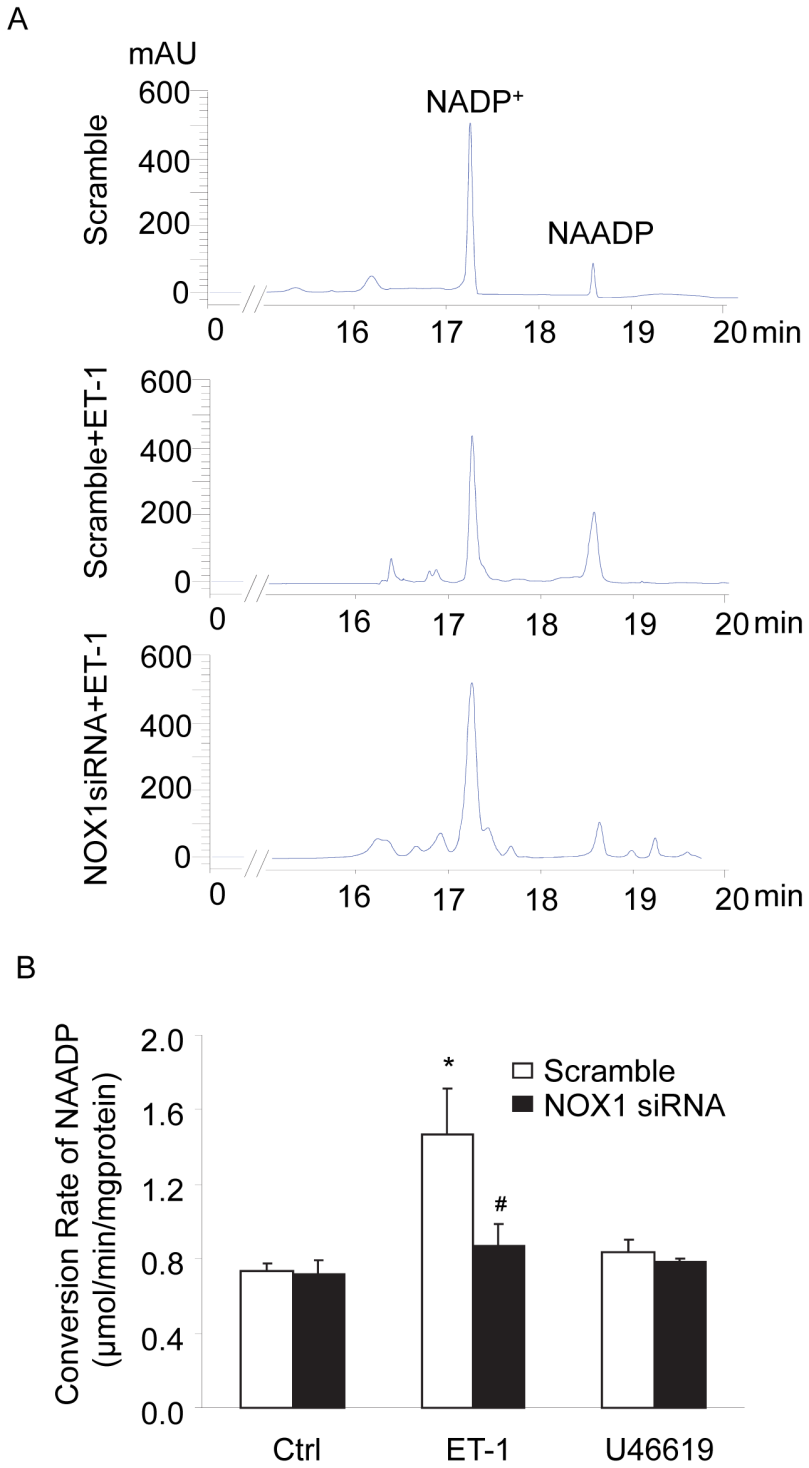
NOX1 activation by ET-1 increased CD38 activity in CAMs. The enzyme activity of CD38 was determined by HPLC analysis of conversion of NADP^+^ into NAADP in CAMs. (A) Typical HPLC chromatograms of NADP^+^ and NAADP in CAMs. (B) Summarized conversion rate showing the effects of NOX1 gene silencing on CD38 activity in CAMs treated with ET-1 or U46619. *P<0.05 *vs.* scramble control; ^#^ *P*<0.05 *vs.* scramble ET-1 (n = 6).

To determine whether NOX1 activation leads to CD38 internalization and thereby activates this signaling enzyme, we measured CD38 activity in CAMs. Since CD38 is the major enzyme for the NAADP synthesis [7], [26], [27], we used HPLC to analyze the conversion rate of NADP^+^ to NAADP as a measure of CD38 activity. Figure 5A is a representative reversed-phase HPLC chromatogram depicting the profile of NADP^+^ and its CD38 enzymatic metabolite, NAADP. Figure 5B summarized the effects of ET-1 and U46619 on NAADP production. ET-1 markedly increased the NAADP conversion rate in CAMs from 0.73±0.043 µmol·min**^−^** ^1^·mg protein**^−^** ^1^ to 1.47±0.247 µmol⋅min**^−^** ^1^·mg protein**^−^** ^1^. This ET-1-induced increase in NAADP conversion was significantly attenuated by NOX1 siRNA. In contrast, U46619 had no effect on the NAADP conversion rate.

### Activation of Rac1-GTPase Increased the Internalization of CD38 in CAMs

**Figure 6:**
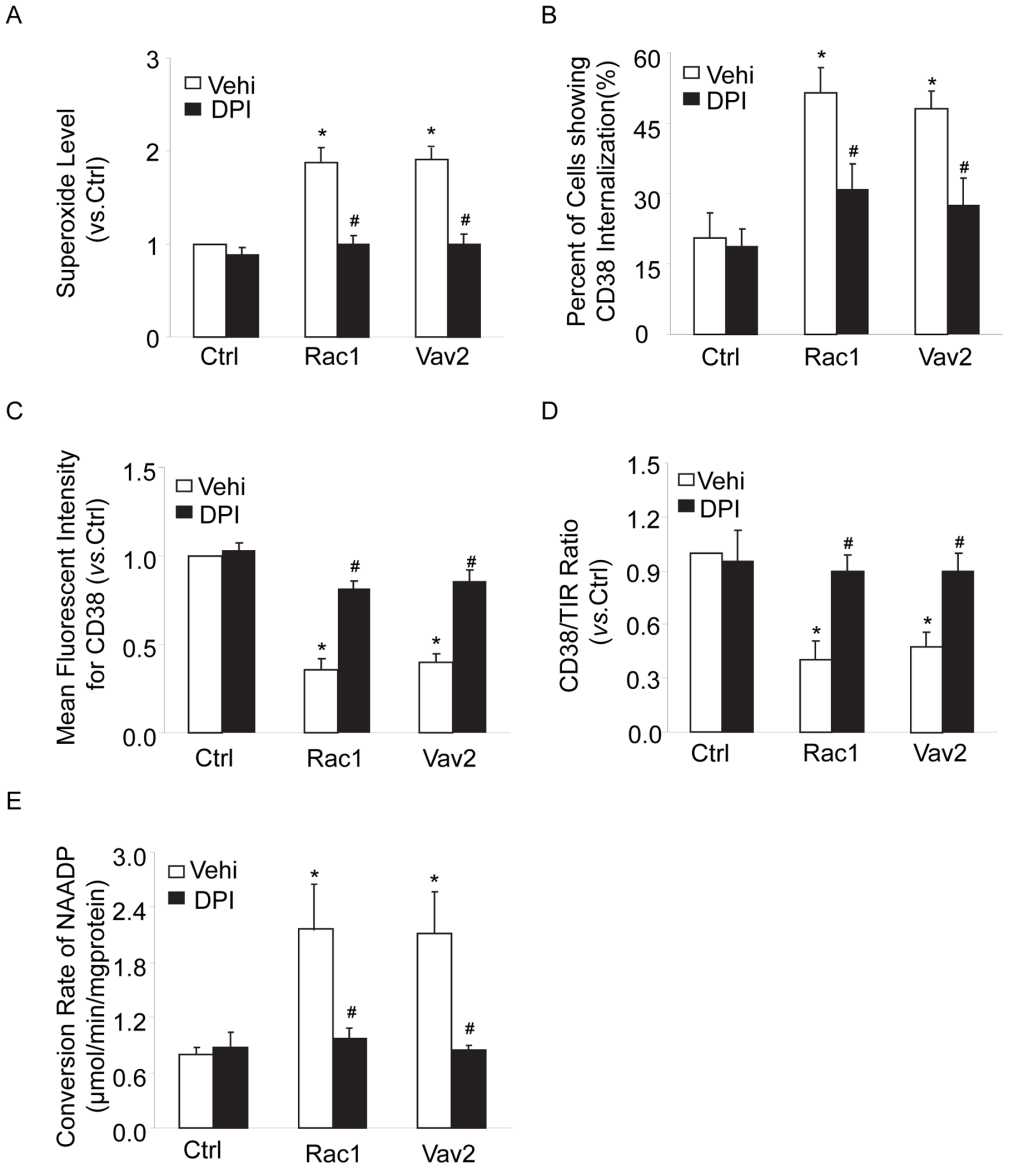
Rac1-GTPase activation by overexpression of Rac1 and Vav2 increased CD38 internalization and activity. Mouse CAMs were transfected with plasmids encoding Rac1 or Vav2 cDNA in the presence or absence of diphenyleneiodonium sulfate (DPI, 50 µM). Then these cells were analyzed for O_2_ **^·−^** production (A), CD38 internalization by confocal microscopy (B), surface CD38 staining in living cells by flow cytometry (C), CD38 expression in the plasma membrane by Western blot (D) and CD38 activity by HPLC (E) as described above in Figure 1–5. Displayed are summarized data from six independent experiments. *P<0.05 *vs.* vehicle control; ^#^ *P*<0.05 *vs.* Rac1 or Vav2 cDNA alone.

We further investigated whether activation of NOX1 by its direct activator, Rac1-GTPase, increases CD38 internalization and activation. Overexpression or increase in Rac1 and Vav2 within cells have been reported to activate NADPH oxidase [28], [29]. In particular, Vav2 as a member of the guanine nucleotide exchange factor-Vav subfamily has been reported to lead to increased Rac1 activity and consequent activation of NADPH oxidase [20]. In the present study, Vav2 was detected in mouse CAMs and increased by 5 folds in Vav2 cDNA-transfected CAMs compared with control, and Rac 1 activity was significantly increased in CAMs transfected with Rac 1 cDNA (such validation data not shown). It was shown that overexpression of Rac1 or Vav2 significantly increased O_2_^·**−**^ production, which was blocked by a non-selective NADPH oxidase inhibitor diphenyleneiodonium sulfate (DPI) (Figure 6A). Correspondingly, CAMs transfected with Rac1 or Vav2 cDNA had significant increases in CD38 internalization (Figure 6B), decreases in CD38 surface staining (Figure 6C), CD38 expression in the plasma membrane (Figure 6D) and increases in CD38 activity (Figure 6E). All these changes were blocked by DPI. ML171 is proved to be a highly selective, cell-permeable, and reversible 2-acetylphenothiazine that is shown to inhibit NOX1 activity. As shown in Figure S2, ML171 effectively reversed the decrease in membrane CD38 protein and increase in O_2_^·**−**^ production in CAMs with the overexpression of Rac1 and Vav2. The results provide further evidence that NOX1 plays a crucial role in mediating endothelin signaling in CAMs.

### Effects of Exogenous ROS on CD38 Internalization in CAMs

**Figure 7:**
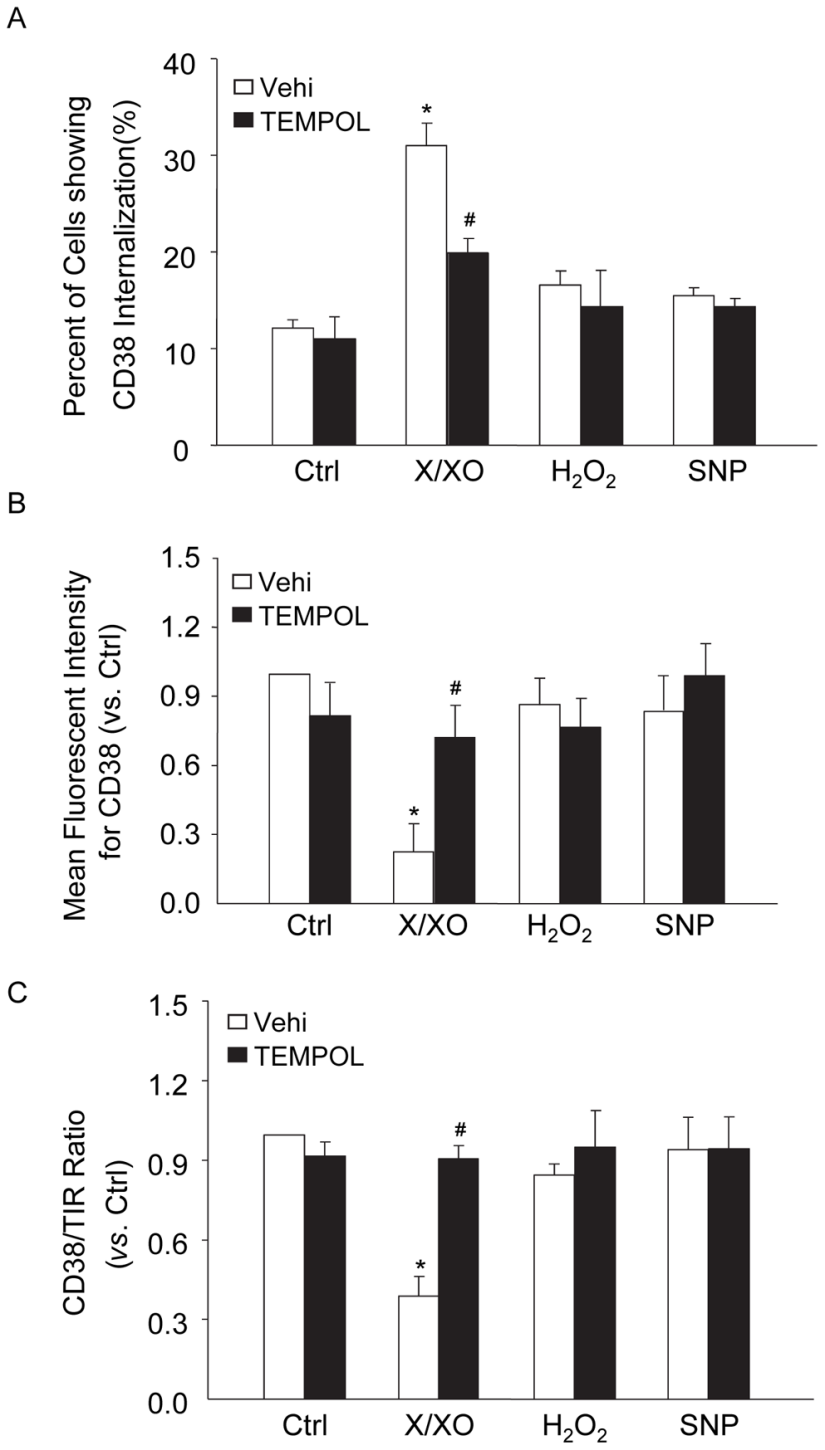
Effects of exogenous O_2_^·−^ on CD38 internalization in CAMs. Mouse CAMs were treated with X/XO (10 µM/0.1 U/ml), hydrogen peroxide (H_2_O_2_, 100 µM) and sodium nitroprusside (SNP, 100 nM) in the presence or absence of O_2_ **^·−^** scavenger 4-hydroxyl-tetramethylpiperidin-oxyl (TEMPOL, 1 mM). Then the cells were analyzed for CD38 internalization (A), surface CD38 staining in living cells (B) and CD38 expression in the plasma membrane (C). Displayed are summarized data from six independent experiments. *P<0.05 *vs.* vehicle control; ^#^ *P*<0.05 *vs.* X/XO alone.

To further confirm the role of extracellular O_2_^·**−**^ in CD38 internalization, we treated cells with the xanthine and xanthine oxidase (X/XO) system to produce exogenous O_2_^·**−**^. As compared with control cells, CD38 internalization was clearly observed in mouse CAMs treated with X/XO (Figure 7A–C). CD38 internalization induced by X/XO-derived O_2_^·**−**^ was inhibited when CAMs were pretreated with cell-permeable O_2_^·**−**^ scavengers 4-hydroxyl-tetramethylpiperidin-oxyl (TEMPOL). These data suggest that it is O_2_^·**−**^ that mediates CD38 internalization and activation. In contrast, neither hydrogen peroxide (H_2_O_2_) nor sodium nitroprusside (SNP), a NO donor, had a significant effect on CD38 internalization (Figure 7A–C).

### Role of MRs Clustering in CD38 Internalization in CAMs

**Figure 8:**
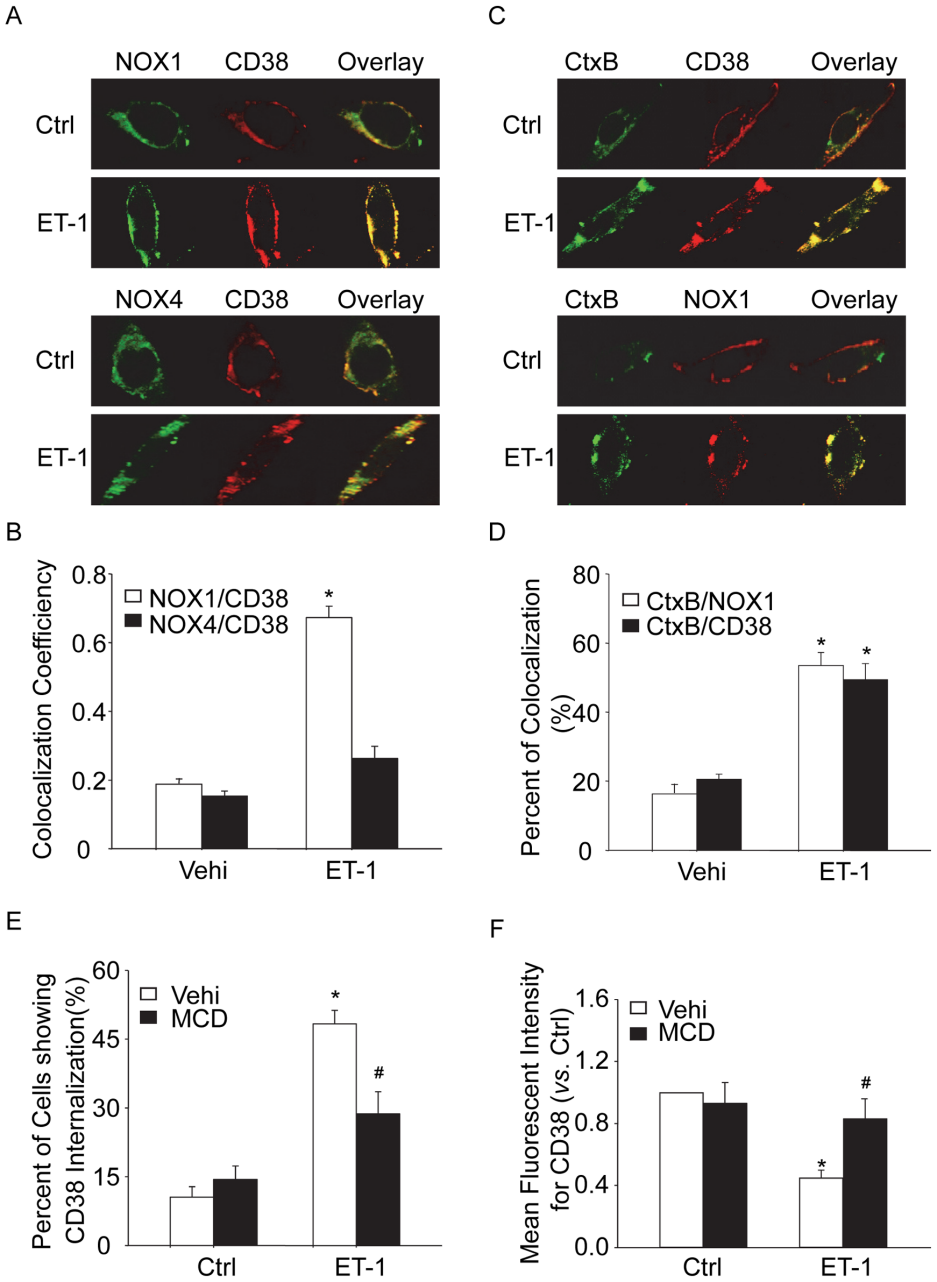
MRs clustering and its role in CD38 internalization of CAMs. (A) Representative confocal fluorescent images showing the colocalization between CD38 and NOX1 or NOX4 in mouse CAMs. (B) Summarized data showing the effects of ET-1 on the colocalization of NOX1 or NOX4 and CD38 in CAMs. (C) Representative confocal fluorescent images showing the colocalization of MRs marker CtxB clustering with CD38 or with NOX1 in mouse CAMs. (D) Summarized data showing the effects of ET-1 on the colocalization between MRs clustering and CD38 or with NOX1 in CAMs. (E) Summarized data showing the effect of MR-disrupting agent MCD (1 mM) on CD38 internalization in CAMs. (F) Summarized data showing the effects of MCD on the mean fluorescent intensity for Alexa488-anti-CD38 staining in CAMs. *P<0.05 *vs.* vehicle control; ^#^ *P*<0.05 *vs.* vehicle ET-1.

To explore the mechanism mediating the action of NOX1 on CD38 internalization or activation, we first observed the spatial location between NOX1 with CD38 in CAMs. The results in Figure 8A and 8B showed that ET-1 significantly induced the colocalization for NOX1 with CD38 in CAMs. In contrast, no significant colocalization was found for NOX4 with CD38 in response to ET-1. These results suggest that NOX1 and CD38 colocalize in special plasma membrane domains upon ET-1 stimulation. To examine the role of MR clustering on CD38 internalization, we further assessed the colocalization of NOX1 or CD38 with a marker for MR, cholera toxin B subunit (CtxB). As shown in Figure 8C and 8D, ET-1 dramatically increased the colocalization of CtxB clustering with CD38 or with NOX1, suggesting that both NOX1 and CD38 became clustered in MRs upon stimulation. In addition, the percentage of cells showing cytosolic expression of CD38 markedly decreased after the pretreatment with methyl-β-cyclodextrin (MCD), a MR*-*disrupting agent (Figure 8E). Using a flow cytometric assay, surface CD38 staining in living CAMs increased in the presence of MCD (Figure 8F). These results suggest that clustered MRs provide membrane signaling platforms to facilitate the spatial translocation of NOX1 with CD38, where O_2_^·**−**^ may be produced to promote CD38 internalization and activation.

## Discussion

The present study for the first time demonstrated that NOX1-derived extracellular O_2_^·**−**^ production is involved in CD38 internalization in CAMs upon ET-1 stimulation. Furthermore, the activation of Rac1 GTPase increased NOX1-derived extracellular O_2_^·**−**^ production and thereby induced CD38 internalization and activation. These results provide direct evidence that Rac1-NOX1-dependent O_2_^·**−**^ production mediates CD38 internalization in CAMs, which represents an important novel mechanism linking receptor activation to CD38 activity in these cells.

Despite increasing evidence that CD38-cADPR/NAADP signaling is an essential mechanism in the regulation of the intracellular Ca^2+^ levels in a variety of mammalian cells [4], [7], [30], [31], so far it remains unknown what mechanism mediates CD38 activation and cADPR/NAADP production in response to various agonists or stimuli and how agonist stimulation couples with CD38 activation to lead to the responses of intracellular effectors. Because CD38 is mainly present on the surface of cells serving as an ecto-enzyme, it is imperative to know how an ecto-enzyme could produce a second messenger cADPR to mediate intracellular Ca^2+^ signaling. In this regard, CD38 internalization has been considered as the prime prerequisite for its enzymatic activation. In the present study, we tested a novel hypothesis that membrane-bound NADPH oxidase NOX1 controls CD38 internalization in CAMs when these cells are stimulated by agonists. ET-1 was used as a CD38 stimulator, which is an important endothelium-derived vasoconstrictor and exerts a wide spectrum of biological effects on smooth muscle cells via ET_A_ and ET_B_ receptors, including inhibition of voltage-gated K^+^ channels, activation of L-type Ca^2+^ channels, as well as mobilization of intracellular Ca^2+^ [32]. Recent studies have indicated that ET-1 mainly exerts its Ca^2+^ mobilizing action in pulmonary arteries through NAADP [33]. Our previous studies have also demonstrated that ET-1 activated CD38 to produce NAADP and NAADP-induced two-phase Ca^2+^ response associated with lysosome Ca^2+^-triggering action and subsequent CICR, which results in a large global increase in [Ca^2+^]_i_. This lysosome-associated Ca^2+^ regulatory mechanism through NAADP has been shown to contribute to the coronary vasoconstrictor response to ET-1. Thus, these previous studies have identified ET-1 as a potent CD38 activator to produce NAADP [7]. The present study explored whether and how ET-1 receptor activation leads to CD38 internalization in CAMs. Using confocal microscopic analysis of CD38 localization, we have clearly shown that ET-1 increased translocation of CD38 into cytosolic compartments, indicating that CD38 internalization occurs in CAMs upon ET-1 receptor activation. Further, this ET-1-induced CD38 internalization was accompanied by subsequent decreases in CD38 expression in the plasma membrane of CAMs as detected by flow cytometry and biotinylation assay of membrane CD38. To our knowledge, these results provide the first evidence that ET-1 receptor activation couples with CD38 internalization in CAMs. This CD38 internalization in CAMs was also observed in response to other stimuli such as Ang II which was blocked by the transfection of NOX1 siRNA (data not shown). It is suggested that NOX1 is necessary for CD38 activation in response to different stimuli or agonists. It seems that the prerequisite for CD38 internalization depends upon whether the agonist use CD38 signaling pathway, which may be associated with receptor linking or clustering into membrane rafts on the cell membrane as we have reported in previous studies [23], [34].

Previous studies have indicated that CD38 activity is stimulated through cell-surface signaling via activation of heterotrimeric G-protein-coupled receptors including endothelin, Ang II, β-adrenergic, and muscarinic receptors [10]. Interestingly, activation of these receptors have been shown to increase vascular NADPH oxidase-dependent O_2_^·**−**^ production, resulting in local oxidative stress and cardiovascular dysfunction [35], [36]. For example, ET-1 has been shown to activate NADPH oxidase via the ET receptor-proline-rich tyrosine kinase-2 (Pyk2)-Rac1 pathway in neonatal ventricular cardiomyocytes [37]. Thus, NADPH oxidase-dependent O_2_^·**−**^ production may represent a novel mechanism initiating CD38 internalization and subsequent signaling cascades. In this regard, we previously demonstrated that NOX1 and NOX4, but not NOX2 are the major NADPH oxidase isoforms in CAMs, which are respectively responsible for extracellular and intracellular O_2_^·**−**^ production [17]. Further, NOX1-derived extracellular O_2_^·**−**^ production is independent of CD38 activity, whereas CD38-Ca^2+^ signaling controls NOX4-derived intracellular O_2_^·**−**^ production [15], [17]. In the present study, we detected NAADP conversion rate by HPLC analysis to reflect CD38 activity and found that NOX1 gene silencing abolished ET-1-induced increases in CD38 activity. Thus, NOX1-derived extracellular O_2_^·**−**^ production is an upstream signaling event of CD38 activation. Consistently, CD38 internalization induced by ET-1 was also markedly inhibited in CAMs when the NOX1 gene was silenced in these cells, whereas silencing of NOX4 gene had no effect on CD38 internalization. Together, these results suggest that NOX1-derived O_2_^·**−**^ may serve in an autocrine-fashion to initiate CD38 internalization and subsequent signaling cascades.

To further investigate whether NOX1-dependent O_2_^·**−**^ plays a triggering role in CD38 internalization, we directly activated NOX1 by enhancement of *Rac* GTPase activity via overexpression of Rac1 or its guanine nucleotide exchange factor Vav2 and then observed CD38 internalization and activation. It is known that NOX1 associates with the membrane subunit p22*^phox^*, which is activated by forming a complex with cytosolic activators p47*^phox^*, p67*^phox^*, and a small *Rac* GTPase [38]. *Rac*1 is one of Rac GTPases, which controls NOX1-dependent O_2_^·**−**^generation in a variety of mammalian cells such as HEK293H cells, in guinea pig gastric mucosal cells, and rat dopaminergic cells [39], [40], [41], [42]. *Rac*1 has been shown to activate NOX1 by anchoring other cytosolic subunits to the transmembrane NOX complex [28], [43], while guanine nucleotide exchange factors (GEFs) facilitate the exchange of GDP for GTP and thus are directly responsible for the activation of *Rac* GTPases [44]. The Vav family is a well-studied subfamily of GEFs regulating the activity of *Rac* GTPases in a variety of mammalian cells [45], [46]. Previous studies by us and others have demonstrated that Vav2 activation caused constitutive upregulation of *Rac*1 resulting in enhancement of ROS production *in vitro* and *in vivo* [47], [48]. In the present study, we found that activation of *Rac*1 by overexpression of *Rac*1 or Vav2 induced extracellular O_2_^·**−**^ production in CAMs, which was blocked by DPI or NOX1 specific inhibitor ML171. These data confirm that *Rac*1 activation causes NOX1 activation and subsequent extracellular O_2_^·**−**^ production in CAMs. Consistently, overexpression of *Rac*1 or Vav2 significantly increased CD38 internalization and activation, which were also blocked when NOX1 was inhibited. These results suggest that not only agonists-induced production of O_2_^·**−**^, but also direct activation of NOX1 leads to CD38 internalization and corresponding activation to produce NAADP in CAMs.

Next, we demonstrated that exogenous administration of extracellular O_2_^·**−**^ also triggered CD38 internalization. By incubation of CAMs with xanthine and xanthine oxidase (X/XO), a typical exogenous O_2_^·**−**^ generating system, we found that this exogenous O_2_^·**−**^ producing system by X/XO induced CD38 internalization, which is consistent with our previous findings that O_2_^·**−**^ from X/XO enhances CD38 activity and its Ca^2+^ signaling in CAMs leading to coronary vasoconstriction [15]. As a comparison, H_2_O_2_ and NO donor **SNP** had no significant effects on CD38 internalization, which is consistent with previous reports where H_2_O_2_ did not significantly alter the ADP-ribosylcyclase activity of CD38 [15] and NO decreased ADP-ribosylcyclase activity in CAMs [49]. Collectively, these data strongly support the view that Rac1-dependent NOX1 activity is responsible for extracellular production of O_2_^·**−**^, which triggers CD38 internalization in CAMs.

**Figure 9:**
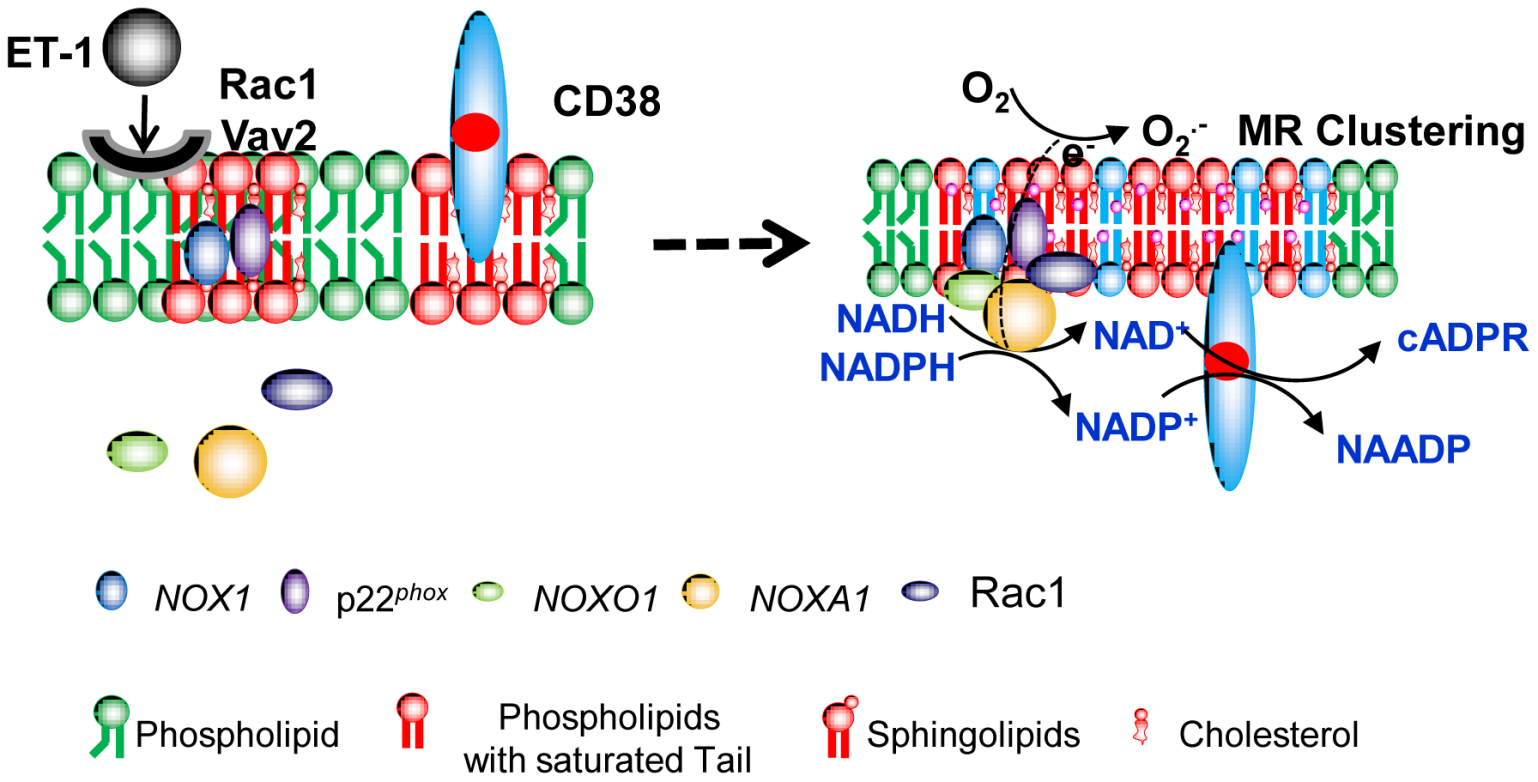
Working model for NOX1-derived redox regulation of CD38 internalization in CAMs. In this model, NOX1-dependent extracellular O_2_^·**−**^ production contributes CD38 internalization. Membrane raft clustering facilitates the spatial translocation and interaction of NOX1-derived O_2_^·**−**^ with CD38 leading to its internalization and exposure of active enzymatic site to its substrate NAD^+^ or NADP^+^.

The present study did not attempt to further explore how NOX1-dependent O_2_^·**−**^ triggers CD38 internalization by binding to or reaction with its domains. It has been demonstrated that CD38 has 12 highly conserved cysteine residues in the extracellular domain, and oxidation of cysteine molecules leads to the formation of one or several disulfide bonds, which may change CD38 protein conformation to trigger CD38 internalization [50]. Further, cysteine 119 and cysteine 201 of CD38 were found to be essential sites for CD38 internalization [14], [50]. Since O_2_ **^−^** ^.^ is a short-lived molecule, the oxidation and activation of CD38 may require NOX1 in proximity with CD38 in the plasma membrane. Therefore, we determined whether a membrane raft (MRs) NOX1 redox signaling platform is formed to cluster CD38 to trigger its internalization and activation through redox modification in the membrane platforms. By confocal microscopy, we observed that ET-1 significantly induced the colocalization between NOX1 and CD38 in CAMs, indicating clustering of both Nox1 and CD38 in MRs platforms. In contrast, no significant colocalization was found between NOX4 and CD38 in respond to ET-1. It appears that NOX1 and CD38 tends to colocalize in special plasma MR domains upon ET-1 stimulation and such spatial organization supports the view that NOX1-derived O_2_^·**−**^ interacts with CD38 in such MR domains, which may oxidize the cysteine residues of CD38 leading to its internalization and activation. Recent studies have reported that MRs are closely associated with the CD38 pathway in various lymphocytes and that these MRs may be responsible for the endocytosis of CD38 [34], [51], [52]. Our previous studies also showed that ceramide-enriched lipid macrodomains contribute to CD38 activity to produce cADPR in CAMs [34] and that MRs platforms mediate the aggregation of membrane **NADPH oxidase** subunits and subsequent activation of this enzyme in bovine coronary arterial endothelial cells [53]. In addition to findings on colocalization of MR marker CtxB clustering with CD38 or with NOX1, we also demonstrated that **methyl-β-cyclodextrin (MCD)**, **MR-disrupting agents** could block CD38 internalization as analyzed by confocal microscopy and flow cytometric assay. We further demonstrated that ET-1 did not induce NOX1 internalization in CAMs (Figure S1). MR disruption by MCD or CD38 gene knockout did not affect membrane expression of NOX1 (Figure S1). These data suggest that NOX1 remain in the cell membrane after triggering CD38 internalization. Recent studies demonstrated that exposure of SMCs to TNF-α result in NOX1 internalization via endocytosis and consequent production of ROS in endosomes, however, thrombin activated NOX1-derived ROS production in SMCs without NOX1 internalization [54]. Thus, NOX1 internalization seems to be dependent on the type of stimulators. Taken together, our data supports a model for redox regulated CD38 internalization (Figure 9) that MRs clustering serves a membrane signaling platform that facilitates the spatial translocation and interaction of NOX1-derived O_2_^·**−**^ with CD38 leading to its internalization and exposure of active enzymatic site to its substrate NAD^+^ or NADP^+^.

In summary, the present study demonstrated a novel mechanism mediating CD38 internalization and activation in CAMs, which is associated with Rac1-NOX1-dependent O_2_^·**−**^ production. MRs clustering facilitates the spatial translocation and interaction of NOX1-derived O_2_^·**−**^ with CD38 and consequently leads to its internalization. These data provide new insights on how NADPH oxidase-mediated redox signaling contributes to the regulation of the CD38 activation and consequent production of intracellular second messengers.

## Supporting Information

Figure S1 **Flow cytometric analysis of surface NOX1 expression in living CAMs.** CAMs from wild-type (CD38^+/+^) and CD38 knockout (CD38^−/−^) mice were treated with ET-1 (100 nM) with or without MR-disrupting agent MCD (1 mM). Cells were then stained with Alexa488 conjugated anti-NOX1 antibodies on ice without fixation and permeablization. The mean fluorescent intensity for Alexa488-anti-CD38 staining were analyzed by flow cytometry (n = 4).
(TIF)

Figure S2 **Effects of NOX1 inhibitor ML117 on CD38 internalization induced by overexpression of Rac1 and Vav2.** Mouse CAMs were transfected with plasmids encoding Rac1 or Vav2 cDNA in the presence or absence of ML117 (100 µM). Then these cells were analyzed for O_2_ **^·−^** production (A) and surface CD38 staining in living cells by flow cytometry (B). *P<0.05 *vs.* vehicle control; ^#^ *P*<0.05 *vs.* Rac1 or Vav2 cDNA alone (n = 4).
(TIF)
