# Supplementary figures and images for: Contribution of NADPH Oxidase to Membrane CD38 Internalization and Activation in Coronary Arterial Myocytes
Source: PLoS One. 2013 Aug 7;8(8):e71212. doi: 10.1371/journal.pone.0071212 (PMC3737089; doi:10.1371/journal.pone.0071212)

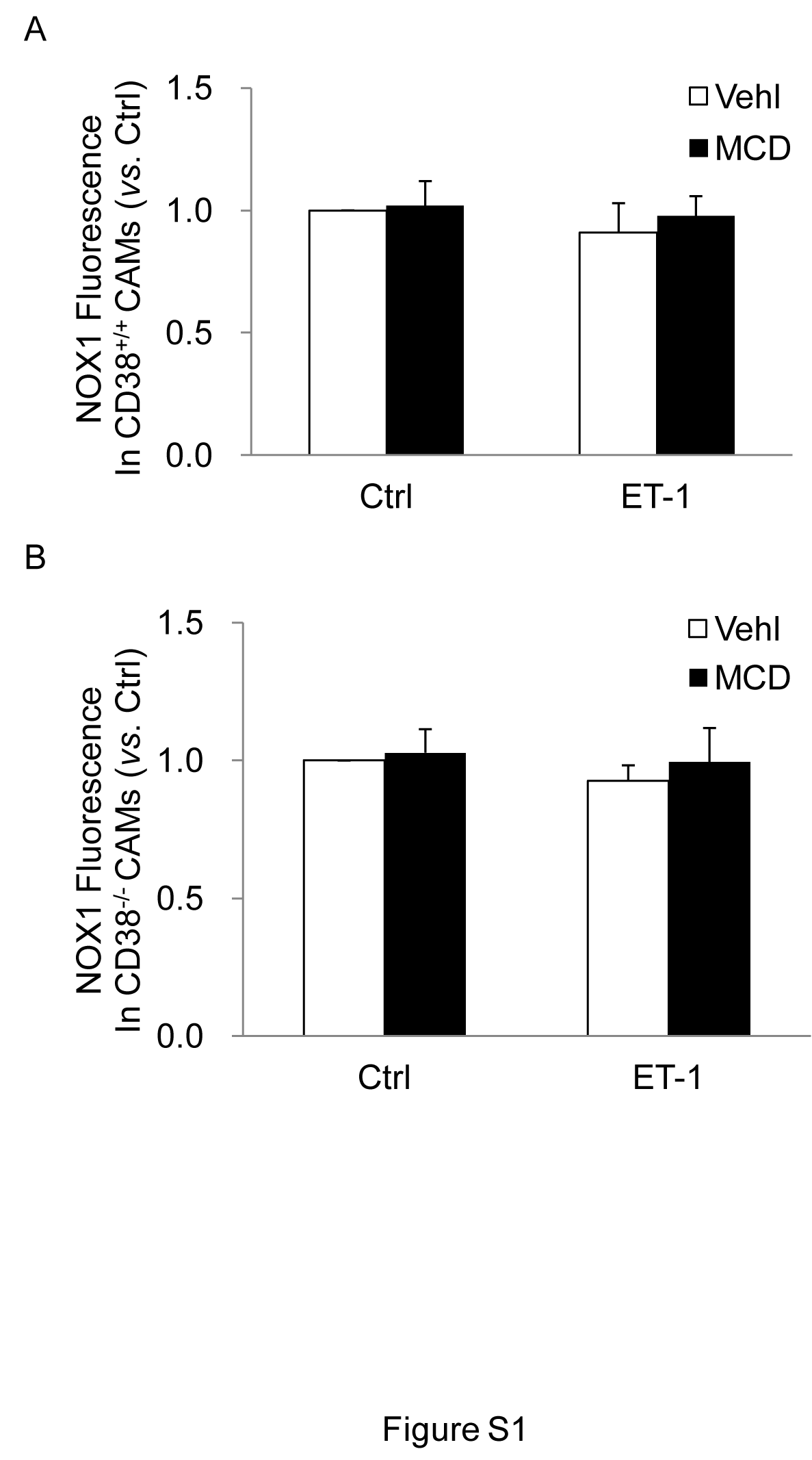

Supplement: Figure S1 — Flow cytometric analysis of surface NOX1 expression in living CAMs. CAMs from wild-type (CD38+/+) and CD38 knockout (CD38−/−) mice were treated with ET-1 (100 nM) with or without MR-disrupting agent MCD (1 mM). Cells were then stained with Alexa488 conjugated anti-NOX1 antibodies on ice without fixation and permeablization. The mean fluorescent intensity for Alexa488-anti-CD38 staining were analyzed by flow cytometry (n = 4). (TIF) [file pone.0071212.s001.tif]

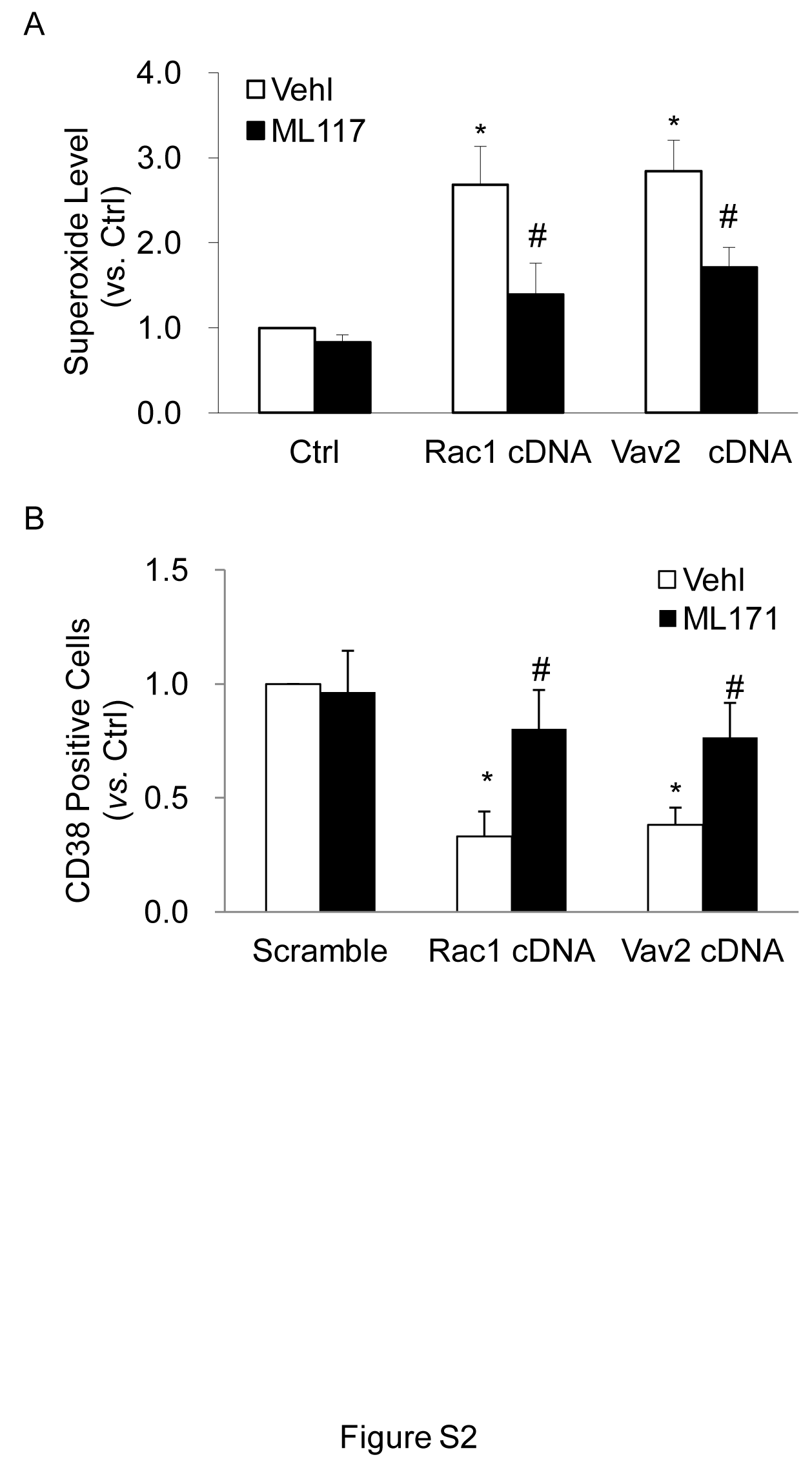

Supplement: Figure S2 — Effects of NOX1 inhibitor ML117 on CD38 internalization induced by overexpression of Rac1 and Vav2. Mouse CAMs were transfected with plasmids encoding Rac1 or Vav2 cDNA in the presence or absence of ML117 (100 µM). Then these cells were analyzed for O2 ·− production (A) and surface CD38 staining in living cells by flow cytometry (B). *P<0.05 vs. vehicle control; # P<0.05 vs. Rac1 or Vav2 cDNA alone (n = 4). (TIF) [file pone.0071212.s002.tif]
